# S1P Increases VEGF Production in Osteoblasts and Facilitates Endothelial Progenitor Cell Angiogenesis by Inhibiting miR-16-5p Expression via the c-Src/FAK Signaling Pathway in Rheumatoid Arthritis

**DOI:** 10.3390/cells10082168

**Published:** 2021-08-23

**Authors:** Chien-Chung Huang, Tzu-Ting Tseng, Shan-Chi Liu, Yen-You Lin, Yat-Yin Law, Sung-Lin Hu, Shih-Wei Wang, Chun-Hao Tsai, Chih-Hsin Tang

**Affiliations:** 1 School of Medicine, China Medical University, Taichung 40402, Taiwan; cchuang10127@mail.cmu.edu.tw (C.-C.H.); cawaii7992@gmail.com (T.-T.T.); chas6119@gmail.com (Y.-Y.L.); nick07202011@yahoo.com.tw (S.-L.H.); 2 Division of Immunology and Rheumatology, Department of Internal Medicine, China Medical University Hospital, Taichung 40402, Taiwan; 3 Department of Medical Education and Research, China Medical University Beigang Hospital, Yunlin 65152, Taiwan; sdsaw.tw@yahoo.com.tw; 4 Institute of Medicine, Chung Shan Medical University, Taichung 40201, Taiwan; andrewlaw@gmail.com; 5 Department of Orthopedics, Chung Shan Medical University Hospital, Taichung 40201, Taiwan; 6 Department of Family Medicine, China Medical University Hsinchu Hospital, Hsinchu 30210, Taiwan; 7 Institute of Biomedical Sciences, Mackay Medical College, New Taipei City 25245, Taiwan; shihwei@mmc.edu.tw; 8 Department of Medicine, Mackay Medical College, New Taipei City 25245, Taiwan; 9 Graduate Institute of Natural Products, College of Pharmacy, Kaohsiung Medical University, Kaohsiung 80708, Taiwan; 10 Department of Sports Medicine, College of Health Care, China Medical University, Taichung 40402, Taiwan; 11 Department of Orthopedic Surgery, China Medical University Hospital, Taichung 40402, Taiwan; 12 Chinese Medicine Research Center, China Medical University, Taichung 40402, Taiwan; 13 Department of Biotechnology, College of Health Science, Asia University, Taichung 40354, Taiwan

**Keywords:** S1P (sphingosine-1-phosphate), vascular endothelial growth factor, osteoblasts, rheumatoid arthritis

## Abstract

Angiogenesis is a critical process in the formation of new capillaries and a key participant in rheumatoid arthritis (RA) pathogenesis. Vascular endothelial growth factor (VEGF) stimulation of endothelial progenitor cells (EPCs) facilitates angiogenesis and the progression of RA. Phosphorylation of sphingosine kinase 1 (SphK1) produces sphingosine-1-phosphate (S1P), which increases inflammatory cytokine production, although the role of S1P in RA angiogenesis is unclear. In this study, we evaluated the impact of S1P treatment on VEGF-dependent angiogenesis in osteoblast-like cells (MG-63 cells) and the significance of SphK1 short hairpin RNA (shRNA) on S1P production in an in vivo model. We found significantly higher levels of S1P and VEGF expression in synovial fluid from RA patients compared with those with osteoarthritis by ELISA analysis. Treating MG-63 cells with S1P increased VEGF production, while focal adhesion kinase (FAK) and Src siRNAs and inhibitors decreased VEGF production in S1P-treated MG-63 cells. Conditioned medium from S1P-treated osteoblasts significantly increased EPC tube formation and migration by inhibiting miR-16-5p synthesis via proto-oncogene tyrosine-protein kinase src (c-Src) and FAK signaling in chick chorioallantoic membrane (CAM) and Matrigel plug assays. Infection with SphK1 shRNA reduced angiogenesis, articular swelling and cartilage erosion in the ankle joints of mice with collagen-induced arthritis (CIA). S1P appears to have therapeutic potential in RA treatment.

## 1. Introduction

Angiogenesis is a critical driver of rheumatoid arthritis (RA) development [1], as are endothelial progenitor cells (EPCs) [2,3], which contain the cell surface markers CD34, CD133 and vascular endothelial growth factor receptor 2 (VEGFR2), which stimulate postnatal vasculogenesis [4] and angiogenic function [5]. VEGF induces EPC proliferation and migration, and facilitates angiogenesis [5], enabling the development of RA [6,7]. We have previously demonstrated that the proinflammatory cytokine cysteine-rich 61 (CCN1) stimulates VEGF expression in osteoblasts and upregulates EPC angiogenesis in RA [7]. EPC-dependent angiogenesis therefore seems to be a worthwhile treatment target in RA.

The platelet-derived lysophospholipid mediator sphingosine-1-phosphate (S1P) inhibits platelet-derived growth factor-promoted chemotaxis and activates cellular Ras-related C3 botulinum toxin substrate (Rac), which are implicated in RA pathogenicity [8]. Various biologic functions that are regulated by the S1P/S1P receptor axis include tumor invasiveness and progression, angiogenesis, vasculogenesis, skeletal muscle and nervous system degeneration [9,10,11]. Research has revealed that S1P/S1P receptor 1 (S1P_1_) signaling can regulate osteoimmunology [12], and the S1P receptor is upregulated in synovial tissue from the collagen-induced arthritis (CIA) mouse model [13]. Proangiogenic factors can stimulate the sphingosine kinase 1 (SphK1)/S1P/S1P_1_ pathway to upregulate fibroblast-like synoviocyte (FLS) proliferation and migration and facilitate angiogenesis in a rat model of RA [14]. Moreover, inflammation induction increases in S1P/S1P_3_ signaling and stimulates increased production of interleukin (IL)-6 in FLSs from CIA mice [13]. We have previously shown that S1P upregulates the expression of IL-1β and IL-6 in human osteoblasts [15,16], which suggests that S1P may serve as an inflammatory mediator during arthritis progression.

Osteoblasts are important in RA cartilage pathology [7,17]. The secretion of proinflammatory cytokines by osteoblasts facilitates RA development [17,18], and upregulated IL-17 expression in osteoblasts triggers the migration of monocytes in RA disorders [19]. In addition, osteoblast-synthesized VEGF enhances EPC angiogenesis during RA progression [7], while we have previously demonstrated that S1P stimulates IL-1β production through the JAK and STAT3 signaling pathways in osteoblasts [20], and the S1P/S1P receptor axis reportedly promotes cyclooxygenase-2 and prostaglandin E2 in RA [21]. Previous research has shown that microRNAs (miRNAs) regulate the progression of RA [22,23], and we have reported miRNA involvement in inflammation and angiogenesis [16,24,25]. Notably, S1P can regulate miRNA expression in human disorders such as cancer and rheumatic disease [26,27,28]. However, research has yet to clarify the role of S1P in RA angiogenesis. Our study has identified higher S1P and VEGF expression in patients with RA than those with osteoarthritis (OA). S1P treatment increased the production of osteoblast-derived VEGF and facilitated EPC-driven angiogenesis by blocking miR-16-5p synthesis through the c-Src/FAK signaling pathway. Inhibition of S1P expression diminished VEGF-dependent angiogenesis and reduced CIA in mice. S1P is therefore worth targeting in RA.

## 2. Materials and Methods

### 2.1. Materials

Antibodies against the phosph- FAK (catalog number: sc-374668) and c-Src (catalog number: SC-12928-R), total protein of c-Src (catalog number: SC-5266) and FAK (catalog number: SC-1688), VEGF (catalog number: SC-7269), SphK1 (catalog number: sc-365401) and β-actin (catalog number: sc-47778) were bought from Santa Cruz Biotechnology (Dallas, TX, USA). Small interfering RNAs were obtained from Dharmacon™ (Lafayette, CO, USA). Control mimic and miRNA mimic were provided by Thermo Fisher Scientific (Waltham, MA, USA). The TaqMan^®^ MicroRNA Reverse Transcription Kit, qPCR primers and detection probes were all obtained from Applied Biosystems (Waltham, MA, USA). The Src inhibitor (10 µM PP2, catalog number: P0042) was supplied by Sigma-Aldrich (St. Louis, MO, USA) and the FAK inhibitor (10 µM FAKi, catalog number: 324878) by Calbiochem (San Diego, CA, USA).

### 2.2. The Collection of Synovial Fluids

The study was granted approval by the Institutional Review Board (IRB) of China Medical University Hospital (CMUH, Taichung, Taiwan). Each patient provided written informed consent before participating in the research. Synovial fluid samples were collected during total knee arthroplasty.

### 2.3. Cell Culture

Human osteoblast-like cells (MG-63) were purchased from the American Type Cell Culture (ATCC, Manassas, VA, USA) and cultured in Eagle’s Minimum Essential Medium (MEM) supplemented with 50 U/mL penicillin, 50 µg/mL streptomycin and 10% fetal bovine serum (FBS), then maintained in a cell culture incubator at 37 °C in 5% CO_2_. Human EPCs were isolated and cultured following the method detailed in our published reports [29,30].

### 2.4. Immunoblotting Analysis

Cell lysates were separated by 8–10% SDS-PAGE and transferred onto Immobilon^®^ PVDF membranes (Millipore, Billerica, MA, USA). Immunoblotting data are shown according to the procedures detailed in our past investigations [31,32,33].

### 2.5. Quantitative Real-Time PCR (qPCR) Analysis 

TRIzol^®^ reagent (MDBio Inc., Taipei, Taiwan) was used to extract RNA from the osteoblasts and the oligo(dT) primers were used to reverse transcribe messenger RNA (mRNA) to complementary DNA (cDNA). SYBR^TM^ Green Master Mix (Applied Biosystems, Waltham, MA, USA) was used for real-time quantitative polymerase chain reaction (qPCR) analysis. [34,35,36]. The real-time PCR oligo(dT) primers used for genotyping were as follows: VEGF (forward), 5′-AGGGCAGAATCATCACGAAGT′; VEGF (reverse), 5′-AGGGTCTCGATTGGATGGCA-3′; GAPDH (forward), 5′-ACCACAGTCCATGCCATCAC -3′; GAPDH (reverse), 5′-TCCACCACCCTGTTGCTGTA-3′; miR-16-5p, 5′-TAGCAGCACGTAAATATTGGCG -3′; U6 (forward), 5′-CGCTTCGGCAGCACATATAC-3; U6 (reverse), 5′-AAAATATGGAACGCTTCACGA-3′.

### 2.6. ELISA Assay

S1P and VEGF expression in the synovial fluid of patients was quantified by an ELISA kit (R&D Systems, Inc., Minneapolis, MN, USA). Conditioned medium (CM) was collected from the osteoblasts after 24 h of S1P treatment and quantified for VEGF expression using the ELISA kit, following the manufacturer’s instructions.

### 2.7. EPC Migration and Tube Formation 

Migration activity was examined by Transwell plates (8 μM pores) (Costar/Corning, Lowell, MA, USA). We seeded 1 × 104 EPCs into the upper chamber in 200 μL of 10% FBS in MV2 complete medium. The lower chamber contained 150 μL of 20% FBS in MV2 complete medium and 150 μL CM. After 24 h, cells were fixed in 3.7% formaldehyde solution and stained with crystal violet (0.05%) for 15 min. Cells on the upper side of the filters were removed and washed with PBS. Cells on the undersides of the filters were examined and counted under microscope (200x magnification) (Nikon H600L, Nikon, Tokyo, Japan). Each clone was plated three times for each experiment, and each experiment was repeated at least three times [37]. Tube formation methodology followed our previous research [37]. Matrigel (BD Biosciences, Bedford, MA, USA) was dissolved at 4 °C, then added at a concentration of 200 µL to each well of 24-well plates and incubated at 37 °C for 15 min. Briefly, EPCs were resuspended in MV2 serum-free medium and mixed with CM from the S1P-treated MG63 cells, then added to the wells. After 6–8 h of incubation at 37 °C, EPC tube formation was examined by microscopy. Briefly, this examination focused on 5 areas that were dense with active tube formation. For the migration and tube formation assays, VEGF protein (5 μg/mL) (R&D Systems, Inc., Minneapolis, MN, USA) was used as a positive control and IgG protein (5 μg/mL) (R&D Systems, Inc., Minneapolis, MN, USA) as a negative control; VEGF antibody (5 μg/mL) (R&D Systems, Inc., Minneapolis, MN, USA) was used to reduce S1P-induced increases in VEGF protein expression. Tube branches and tube lengths were examined at a magnification of 20x and calculated with MacBiophotonics ImageJ software.

### 2.8. Transfection of Small Interfering RNAs and miRNA Mimic

MG63 cells were transfected with siRNAs (control, S1P1, S1P3, FAK, Src) or miR-16-5p mimic for 24 h using Lipofectamine 2000 (Invitrogen, Waltham, MA, USA), as described in our previous study [38].

### 2.9. The Chick Chorioallantoic Membrane Assay

The chicken chorioallantoic membrane (CAM) system assessed angiogenic activity, as described previously [7,39]. Fertilized chick embryos were incubated in an 80% humidified atmosphere at 37 °C.

### 2.10. Matrigel Plug Assay

For the Matrigel plug assay, we used 10 mice in each group and subcutaneously injected them with 0.2 mL Matrigel mixture consisting of 50% CM from S1P-treated MG63 cells and 50% Matrigel. After 7 days, we harvested the Matrigel plugs and measured the hemoglobin concentrations, according to previously described methodology [7,18,39].

### 2.11. CIA Mouse Model

CIA immunization was performed according to the methodology detailed in our previous publications [7,16,18]. After receiving two immunizations, the mice received weekly intra-articular injections of ~7.1 × 10^6^ plaque-forming units (PFU) of SphK1 short hairpin RNA (shRNA) or control. Upon sacrifice after 49 days of treatment, phalanges and ankle joints were collected from each mouse then stored in 4% paraformaldehyde for micro-computed tomography (µ-CT) scanning.

### 2.12. Statistical Analysis

All statistical analyses were performed using GraphPad Prism 5.0 (GraphPad Software, San Diego, CA, USA) and all values are presented as the mean ± standard deviation (S.D.). The paired sample *t*-test was used for in vitro analyses of statistical significance; one-way ANOVA followed by Bonferroni testing was used for in vivo analyses.

## 3. Results

### 3.1. Upregulation of S1P and VEGF Expression in RA

**Figure 1:**
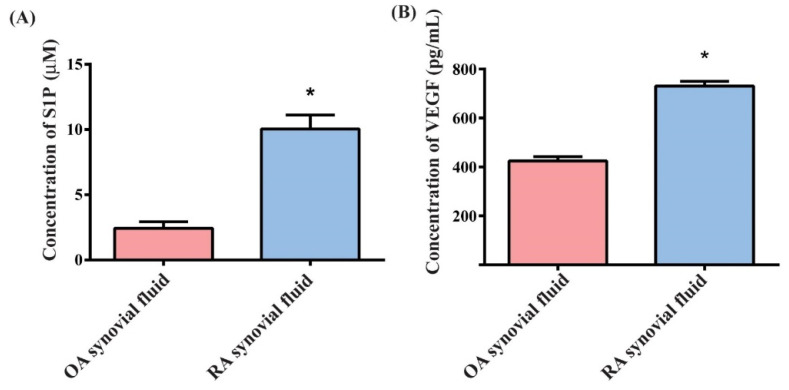
Upregulation of S1P and VEGF expression in RA. S1P (**A**) and VEGF (**B**) levels in human OA and RA synovial fluid were quantified by ELISA. Results are expressed as the mean ± S.D. (*n* = 4). * *p* < 0.05 versus the OA group.

Markedly higher levels of S1P and VEGF expression were identified in samples of RA synovial fluid than in OA samples (Figure 1A,B), suggesting that S1P and VEGF are more critical in RA than in OA.

### 3.2. S1P Facilitates VEGF-Dependent EPC Angiogenesis

**Figure 2:**
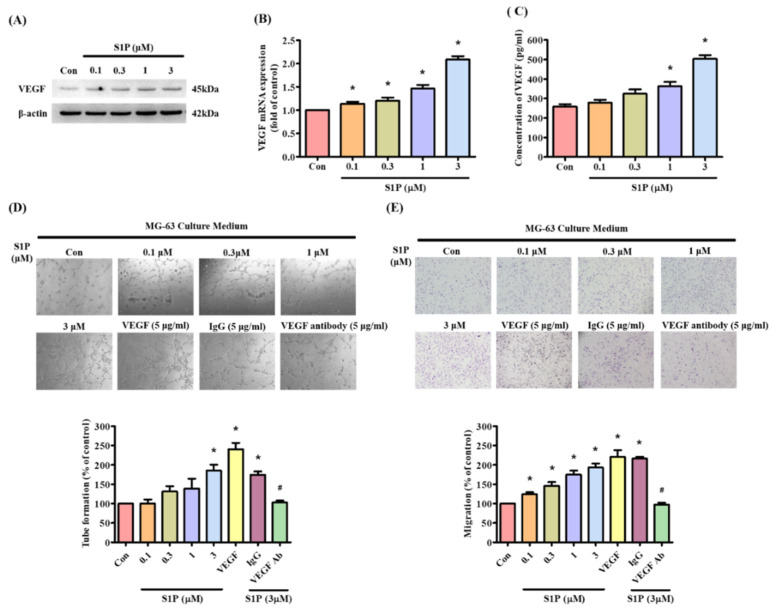
S1P increases osteoblastic VEGF expression and promotes EPC tube formation and migration. (**A**–**C**) MG-63 cells were incubated for 24 h with S1P (0.1–3 μM); VEGF expression was quantified by Western blot, qPCR and ELISA. (**D**,**E**) Collected conditioned medium (CM) was administered to endothelial progenitor cells (EPCs), then EPC angiogenesis was measured by tube formation and the Transwell assay. Results are expressed as the mean ± S.D. (*n* = 3). VEGF protein (5 μg/mL) was used as a positive control and IgG protein (5 μg/mL) as a negative control; VEGF antibody (5 μg/mL) was used to against VEGF protein expression. * *p* < 0.05 versus the control; # *p* < 0.05 versus S1P alone.

Next, we examined whether S1P promotes osteoblastic VEGF expression. Incubation of MG-63 cells with S1P dose-dependently increased VEGF transcription and translation (Figure 2A,B) and the secretion of VEGF protein (Figure 2C). EPC tube formation and migration assays illustrated the effects of S1P-controlled angiogenesis in osteoblasts [6]. CM from S1P-treated MG63 cells significantly increased capillary-like network formation and reorganization as well as EPC migratory activity (Figure 2D,E). Treatment with VEGF antibody dramatically diminished the effects of S1P-treated osteoblast CM on EPC tube formation and migration (Figure 2D,E), indicating that S1P promotes VEGF production in osteoblasts and enhances tube formation and migration of EPCs.

### 3.3. S1P Enhances VEGF-Dependent EPC Angiogenesis through the S1P_1_/S1P_3_ Receptor, c-Src and FAK Pathways

**Figure 3:**
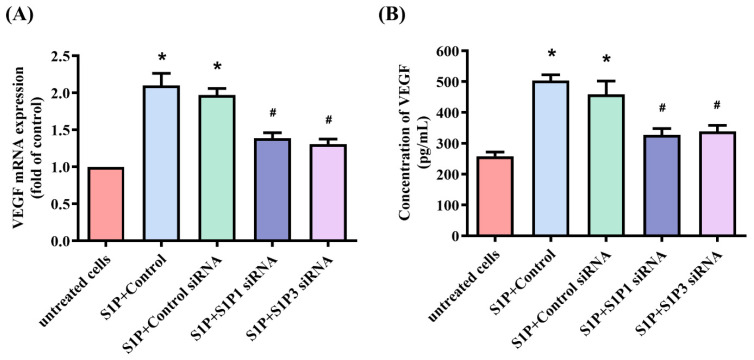
The S1P_1_ and S1P_3_ receptors control S1P-induced upregulation of VEGF expression. Osteoblasts were transfected with S1P_1_ or S1P_3_ siRNAs, before being stimulated with S1P. VEGF levels were quantified by qPCR (**A**) and ELISA (**B**) assays. Results are expressed as the mean ± S.D. (*n* = 3). * *p* < 0.05 versus the control; # *p* < 0.05 versus S1P alone.

**Figure 4:**
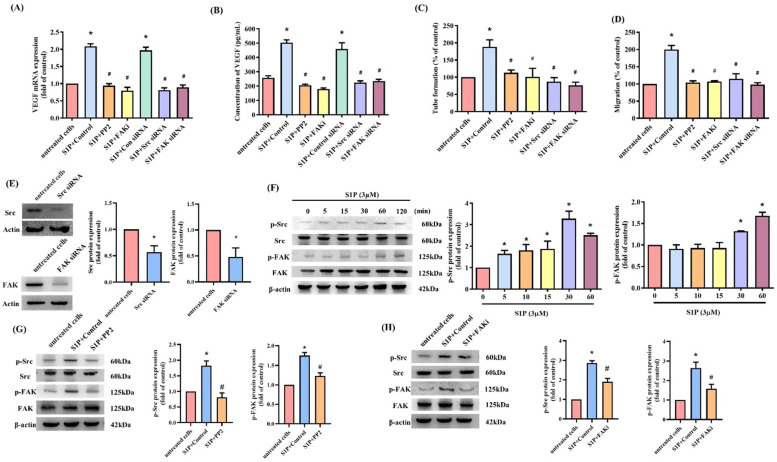
c-Src-dependent FAK activation regulates S1P-induced effects on VEGF production and EPC angiogenesis. (**A**,**B**) Osteoblasts were pretreated with c-Src (PP2) (10 μM) or FAK inhibitors (10 μM), or transfected with siRNAs (c-Src or FAK), then stimulated with S1P. VEGF levels were quantified by qPCR and ELISA. (**C**,**D**) Collected CM was administered to EPCs, and EPC angiogenesis was determined. (**E**) Osteoblasts were transfected with c-Src or FAK siRNAs and Western blot determined c-Src or FAK expression. (**F**) After incubating osteoblasts with S1P, Western blot determined c-Src and FAK phosphorylation. (**G**,**H**) Osteoblasts were pretreated with c-Src (PP2) (10 μM) or FAK inhibitors (10 μM), then stimulated with S1P. Western blot quantified c-Src and FAK phosphorylation and total protein. Results are expressed as the mean ± S.D. (*n* = 3). * *p* < 0.05 versus the control; # *p* < 0.05 versus S1P alone.

S1P_1_/S1P_3_ receptor signaling regulates the participation of S1P in different cellular functions [9,40]. Transfection of osteoblasts with S1P_1_ or S1P_3_ siRNAs antagonized the effects of S1P on VEGF expression (Figure 3). Thus, the S1P_1_ and S1P_3_ receptors mediate the impact of S1P on VEGF production. c-Src and FAK signaling controls different cellular functions, including angiogenesis [29,39]. We therefore sought to determine how c-Src and FAK affect S1P-induced upregulation of VEGF synthesis and angiogenesis. Treatment of osteoblasts with c-Src (PP2) and FAK inhibitors or siRNAs for 24 h reduced the effects of S1P on VEGF mRNA and protein (Figure 4A,B) and inhibited S1P-induced upregulation of EPC tube formation and migration (Figure 4C,D). Transfecting the osteoblasts with c-Src and FAK siRNAs reduced c-Src and FAK expression (Figure 4E), while incubating the osteoblasts with S1P induced c-Src and FAK phosphorylation (Figure 4F). The c-Src inhibitor reversed S1P-induced promotion of c-Src and FAK phosphorylation (Figure 4G,H), whereas the FAK inhibitor did not affect S1P-mediated c-Src phosphorylation, which suggests that S1P promotes VEGF expression and EPC angiogenesis by modulating the c-Src and FAK signaling pathway.

### 3.4. Inhibiting miR-16-5p Controls S1P-Promoted VEGF Synthesis and EPC Angiogenesis

**Figure 5:**
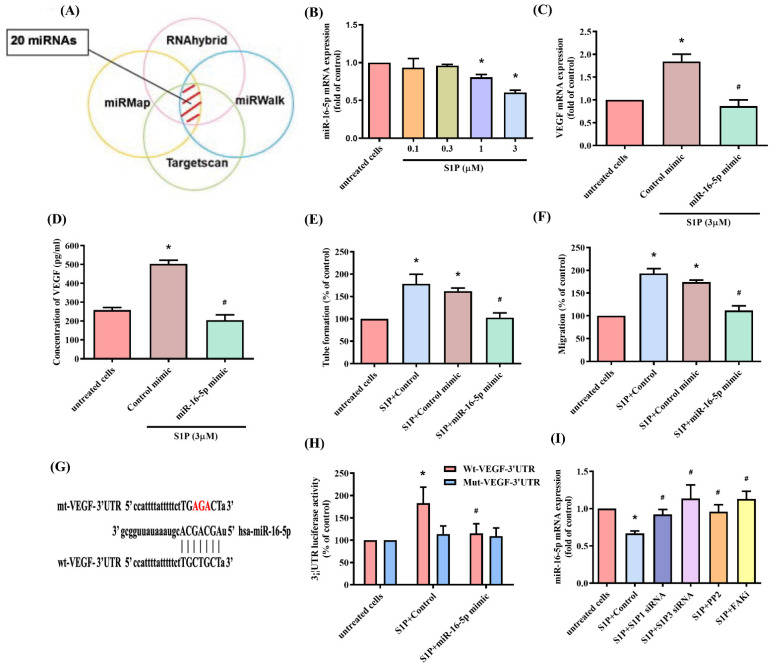
S1P facilitates VEGF synthesis and EPC angiogenesis via the inhibition of miR-16-5p. (**A**) The open-source software enabled identification of miRNAs that possibly disturb the transcription of VEGF. (**B**) Osteoblasts were transfected with S1P. MiR-16-5p expression was determined by the qPCR assay. (**C**,**D**) The osteoblasts were transfected with miR-16-5p mimic, then stimulated by S1P. Levels of VEGF were determined by qPCR and ELISA. (**E**,**F**) Collected CM was administered to the EPCs, and angiogenesis was quantified. (**G**) A schematic representation of human VEGF containing the miR-16-5p binding site. (**H**) The luciferase plasmids with, or without miR-16-5p mimic, were transfected into osteoblasts, before stimulating them with S1P. Measurement of luciferase activity revealed VEGF promoter activity. (**I**) Osteoblasts were transfected with S1P_1_ or S1P_3_ siRNA for 24 h, or pretreated with c-Src or FAK inhibitors for30 min, then stimulated with S1P for 24 h. MiR-16-5p expression was quantified by qPCR. Results are expressed as the mean ± S.D. (*n* = 3). * *p* < 0.05 versus the control; # *p* < 0.05 versus S1P alone.

The dysregulated expression of miRNAs in patients with RA differs from miRNA expression in healthy individuals [41,42]. Using open-source software, we identified 20 miRNAs that potentially interfere with VEGF transcription (Figure 5A). S1P treatment of osteoblasts reduced miR-16-5p synthesis in a concentration-dependent manner (Figure 5B), while transfecting osteoblasts with miR-16-5p mimic antagonized the effects of S1P on VEGF production and EPC angiogenesis (Figure 5C–F). To examine whether miR-16-5p regulates VEGF gene transcription, we constructed luciferase reporter plasmids harboring either the wild-type 3′UTR of VEGF mRNA (wt-VEGF-3′UTR) or a vector containing mismatches in the predicted miR-16-5p binding site (mt-VEGF-3′UTR) (Figure 5G). MiR-16-5p mimic reduced S1P-induced luciferase report activity in the wild-type plasmid only (Figure 5H). Moreover, the S1P_1_ and S1P_3_ siRNAs, as well as the c-Src and FAK inhibitors, all reversed S1P-induced inhibition of miR-16-5p expression (Figure 5I).

**Figure 6:**
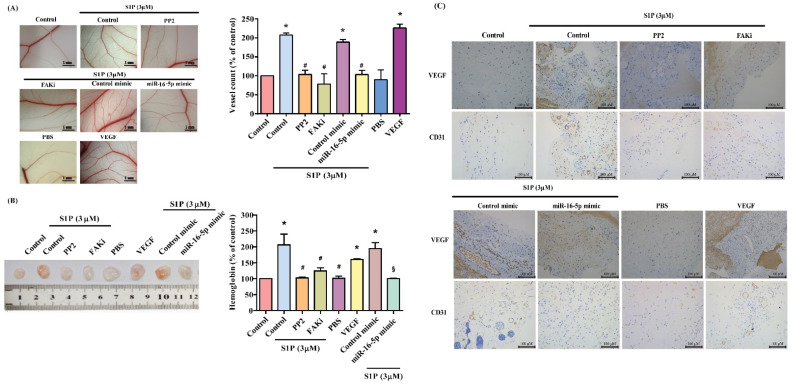
S1P increases angiogenesis in vivo. (**A**) After subjecting osteoblasts to the treatment conditions as indicated, the harvested CM was administered to 6-day-old fertilized chick embryos for 4 days. CAM images were observed by microscopy and photographed, and vessel numbers were counted manually. (**B**) The flanks of nude mice were subcutaneously injected with the Matrigel plugs containing the harvested CM. After 7 days, the plugs were photographed, and hemoglobin levels were evaluated. (**C**) CD31 and VEGF expression were detected on plug specimens by IHC staining. Results are expressed as the mean ± S.D. (*n* = 3). * *p* < 0.05 versus the control; # *p* < 0.05 versus S1P alone.

CAM and Matrigel investigations demonstrated that CM from S1P-treated osteoblasts enhanced vessel formation in vivo (Figure 6A,B), while the c-Src and FAK inhibitors, and miR-16-5p mimic, diminished S1P-promoted induction of vessel formation (Figure 5A,B). The results were confirmed by levels of hemoglobin and the human-specific vessel marker CD31, as well as by VEGF IHC staining (Figure 6C).

### 3.5. Inhibition of S1P Reduces CIA

**Figure 7:**
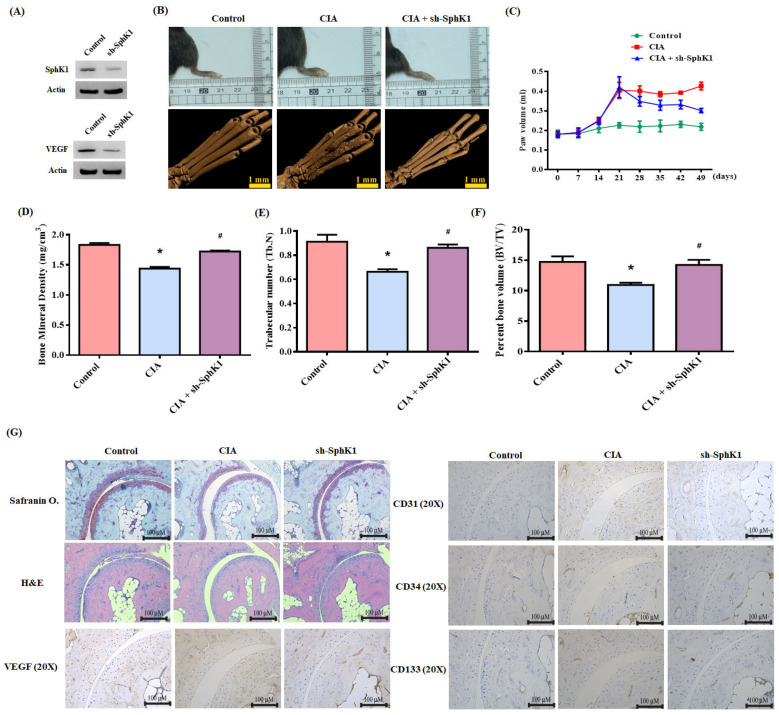
S1P knockdown reduces the in vivo severity of RA. (**A**) After infecting osteoblasts with control or SphK1 shRNA, Western blot determined SphK1 and VEGF expression. CIA mice received intra-articular injections of ~7.1 × 10^6^ PFU SphK1 shRNA or control shRNA on day 14 and were sacrificed on day 49. (**B**) Representative µ-CT images of the hind paws taken on day 49. (**C**) A digital plethysmometer quantified the amounts of hind paw swelling. (**D**–**F**) µ-CT SkyScan Software quantified BMD, trabecular numbers and bone volume. (**G**) Histological sections of ankle joints were stained with H and E or Safranin O/fast green, and immunostained with VEGF, CD31, CD34 and CD133. Results are expressed as the mean ± S.D. (*n* = 3). * *p* < 0.05 versus the control group; # *p* < 0.05 versus S1P alone.

SphK1 shRNA was used to validate the in vivo role of S1P. Infection of MG-63 cells with SphK1 shRNA reduced SphK1 and VEGF expression (Figure 7A). Compared with controls, CIA mice exhibited significant paw swelling that improved after administration of SphK1 shRNA (Figure 7B,C). Micro-CT imaging of the hind paws revealed that SphK1 shRNA reversed CIA-induced reductions in bone mineral density (BMD), trabecular (Tb) number and bone volume (BV) (Figure 7D–F). According to IHC staining data, levels of CD31, CD34, CD133 and VEGF expression were markedly higher in CIA mice than in controls. Notably, SphK1 shRNA treatment antagonized CD31, VEGF, CD34 and CD133 expression (Figure 6G). These results indicate that inhibiting S1P lowers EPC expression and disease activity in CIA.

## 4. Discussion

RA is well recognized for its considerable impact on synovial inflammation and joint destruction [43,44,45]. The development of RA relies upon pannus formation and neovascularization [1], as well as VEGF-induced stimulation of angiogenesis [6]. We have previously documented S1P-induced increases in the expression of IL-6 and IL-1β in human osteoblasts [15,21]. In this study, we observed much higher S1P and VEGF expression in RA synovial fluid compared with OA samples, emphasizing the importance of S1P and VEGF in the pathogenesis of RA, which is reinforced by our evidence showing that S1P stimulates osteoblastic VEGF production and facilitates EPC angiogenesis by blocking miR-16-5p synthesis via c-Src/FAK signaling.

EPCs also stimulate new vessel formation [2,3]. Promotion of EPC mobilization by tumor-secreted VEGF facilitates tumor development and angiogenesis [46]. EPC angiogenesis plays a vital role in RA [6,47]. EPC infiltration into joints has been reported in RA [6]. Compared with synovial fluid from healthy individuals, RA synovial fluid facilitates EPC infiltration and angiogenesis [6], indicating that EPC-dependent angiogenesis is an important step during RA progression. In this study, CM from S1P-treated osteoblasts increased EPC angiogenesis. Our CIA animal model confirmed higher levels of EPC-specific markers compared with levels in healthy controls. SphK1 shRNA inhibited S1P expression, which reduced levels of VEGF expression and EPCs, and mitigated the severity of RA. Thus, inhibition of S1P shows promise as a novel strategy in RA, reducing EPC angiogenesis and disease development.

Successful bone formation depends upon osteoblasts forming and maintaining the skeletal architecture [48,49]. Osteoblasts are critical regulators of arthritis progression [50,51]. Several proinflammatory cytokines, including IL-1β, IL-6 and oncostatin M, are produced by osteoblasts in the joint microenvironment [15,21]. Osteoblast production of monocyte chemoattractant protein 1 triggers the migration and adhesion of monocytes to the RA synovial membrane, stimulating disease progression [52]. Importantly, synthesis of IL-18 and VEGF by osteoblasts promotes angiogenesis during RA [25], indicating that osteoblast-mediated angiogenesis has a critical role in the development of this disease. Our cellular model revealed that S1P increases VEGF mRNA and protein expression in osteoblasts. CM from S1P-treated osteoblasts also facilitated angiogenesis in EPCs, confirming the role of osteoblast-regulated EPC angiogenesis in RA.

c-Src/FAK signaling activation is essential for regulating various cellular functions [53]. Our investigations found that c-Src and FAK inhibitors reduced S1P-enhanced VEGF expression in osteoblasts and EPC angiogenesis. This was confirmed by findings from genetic siRNA experiments demonstrating that c-Src and FAK mediate the angiogenic effects of S1P. Treatment of osteoblasts with S1P augmented c-Src and FAK phosphorylation, while c-Src inhibitor treatment inhibited S1P-promoted FAK phosphorylation. This suggests that FAK is a downstream molecule of c-Src, although FAK has been reported to be an upstream mediator of c-Src [39]. In our study, the lack of any effect from a FAK inhibitor on S1P-facilitated phosphorylation of c-Src indicates that FAK activation depends upon c-Src and that this pathway controls S1P-induced VEGF expression and EPC angiogenesis in human osteoblasts.

MiRNAs post-transcriptionally regulate gene expression [54]. In RA, aberrant miRNA expression regulates the expression of inflammatory pathways [41,42]. In this study, stimulation of osteoblasts with S1P inhibited miR-16-5p expression and transfecting them with miR-16-5p mimic antagonized S1P-promoted upregulation of VEGF expression and EPC angiogenesis. Treating the osteoblasts with c-Src and FAK inhibitors reversed S1P-promoted inhibition of miR-16-5p expression, suggesting that S1P may increase VEGF and EPC angiogenesis by blocking miR-16-5p production via c-Src/FAK signaling.

## 5. Conclusions

**Figure 8:**
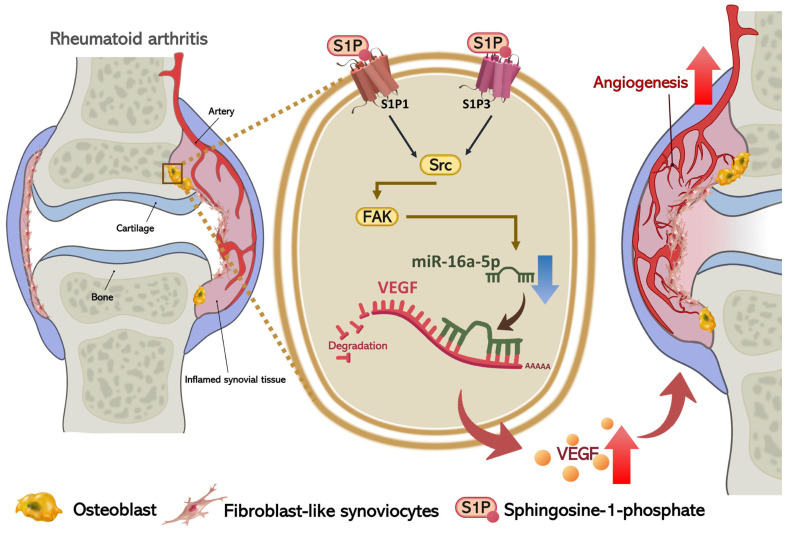
Schematic diagram summarizes the mechanisms of S1P-induced EPC angiogenesis during RA pathogenesis. S1P induces VEGF expression in osteoblasts by suppressing miR-16-5p expression via the S1P_1_ and S1P3 receptors, and the c-Src and FAK signaling pathways, and promotes EPC angiogenesis in RA.

In conclusion, we have determined that S1P increases osteoblastic VEGF expression and subsequently facilitates EPC angiogenesis by blocking miR-16-5p synthesis through c-Src/FAK signaling (Figure 8). The evidence supports the targeting of S1P in RA treatment regimens.

## Data Availability

The raw data for this study are available from the corresponding authors.
